# 5PSeq Explorer: interactive analysis of co-translational mRNA decay and ribosome dynamics

**DOI:** 10.1080/15476286.2026.2639616

**Published:** 2026-03-06

**Authors:** Irene Stevens, Vicent Pelechano

**Affiliations:** Science for Life Laboratory (SciLifeLab), Department of Microbiology, Tumor and Cell Biology (MTC), Karolinska Institutet, Stockholm, Sweden

**Keywords:** Ribosome dynamics, mRNA decay, 5PSeq, degradome sequencing, codon optimality

## Abstract

Co-translational mRNA decay occurs when 5’to3’ exonucleases follow the last translating ribosome, generating *in vivo* ribosome protected fragments. Degradome sequencing (5PSeq)therefore offers unique insights into ribosome dynamics. Despite its potential, resources for systematic analysis of 5′P mRNA decay intermediates and associated features, such as ribosome stalls and collisions, are scarce. We introduce 5PSeq Explorer, a web-based platform built from 773 uniformly processed 5PSeq datasets across 23 species in bacteria and Ascomycota suitable for exploring ribosome dynamics in vivo at codon, amino acid, and transcript levels. By integrating normalized counts, structured metadata, and scalable visualization tools, 5PSeq Explorer provides a framework for studying the crosstalk between mRNA decay and ribosome dynamics. To ensure reproducibility and accessibility, we offer both a public web interface and a Docker-based plug-and-play local version. URL: https://fivepseq-explorer.serve.scilifelab.se/app/fivepseq-explorer.

## Introduction

The translation of messenger RNA (mRNA) is a central step in gene expression. However, the presence of mRNA molecules alone does not guarantee active protein synthesis. Cells tightly regulate the transcription, translation, and degradation of mRNAs. Regulation of mRNA life after synthesis (post-transcriptional regulation) is critical for rapid (i.e. within minutes) adaptations to environmental perturbations. Importantly, translation and mRNA stability are not independent processes, but rather directly interconnected [1].

Alterations in ribosome dynamics, such as codon-specific ribosome stalls [2], ribosome collisions, frameshifts [3] and codon optimality [4] can directly modulate mRNA lifespan and protein synthesis. Genome-wide studies of ribosome dynamics commonly rely on ribosome profiling, which captures ribosome-protected fragments (RPFs) after *in-vitro* RNA digestion [5–7] followed by high-throughput sequencing. To facilitate access to the growing number of ribosome profiling datasets (>14,000 samples), several data portals have been implemented, such as Riboseq.org [8], TranslatomeDB [9] and RPFdb (Ribosome Profiling Database) [10]. These platforms re-processed publicly available ribosome profiling data and often incorporate tools for visualizing ribosome dynamics at transcript (Trips-Viz, a transcript browser of ribosome profiling data [11,12]) and genome-wide levels (GWIPS-viz, Genome Wide Information of Protein Synthesis [13,14], RiboGalaxy [15,16]). These tools have proven useful in the discovery of new translated regions [17–19] and giving investigators detailed views of translation events at sites of interest [20,21]. However, since ribosome profiling focuses on the bulk of soluble ribosomes, it can sometimes obscure the behaviour of ribosome alterations leading to RNA decay.

An alternative approach, 5′P degradome sequencing (5PSeq), exploits the fact that mRNA decay occurs co-translationally. Specifically, 5′→3′ exonucleases can trail the last elongating ribosome, generating decay intermediates that reflect ribosome positions *in vivo* [3,22,23]. Thus, the presence of 5′ monophosphorylated mRNA decay intermediates can provide information regarding ribosome position *in vivo*. Mapping these fragments provides nucleotide-resolution insights into ribosome stalls and collisions in yeast (*Ascomycota*), plants, and bacteria with 5’-3’ RNA exonucleases (*RNase J*) [24]. While 5PSeq is not suited for estimating global translation rates, its technical simplicity and ability to capture co-translational events associated to mRNA decay [3,25] makes it a powerful tool for studying ribosome dynamics under diverse conditions [26]. Despite this potential, resources for systematic exploration of 5PSeq data remain limited. Our lab has previously developed a computational analysis pipeline, FivePseq [27] to facilitate the reproducible analysis of degradome datasets. However, its scope was limited to small batches.

Here, we present 5PSeq Explorer, a web-based platform dedicated to visualizing and comparing 5PSeq-derived ribosome dynamics across species and perturbations. This resource aggregates 773 uniformly processed 5PSeq datasets spanning 23 species. By integrating structured metadata, normalized counts and scalable visualization tools, 5PSeq Explorer provides a comprehensive framework for investigating the interplay between mRNA decay and translation dynamics. A public web interface and a Docker-container local version ensure reproducibility and accessibility for the research community.

## Results

### Data collection and processing

We compiled 773 5PSeq datasets from 18 published studies (Supplementary Table S2) interrogating various aspects of biology at the interface of mRNA decay and translation, including both genetic and environmental perturbations. For Eukaryotes, the collection consists of 376 yeast samples (*Ascomycota* phylum) with an overrepresentation of *Saccharomyces* cerevisiae (*n* = 338). For bacteria we provide 397 samples across *Actinomycetota*, *Bacillota*, *Bacteroides*, *Bacteroidota*, *Proteobacteria*, *Pseudomonadota* and *Verrucomicrobiota* phyla with an overreprepresentation for *Bacillus subtilis* (*n* = 67) and *Aggregatibacter actinomycetemcomitans* (*n* = 59).

**Figure 1.**
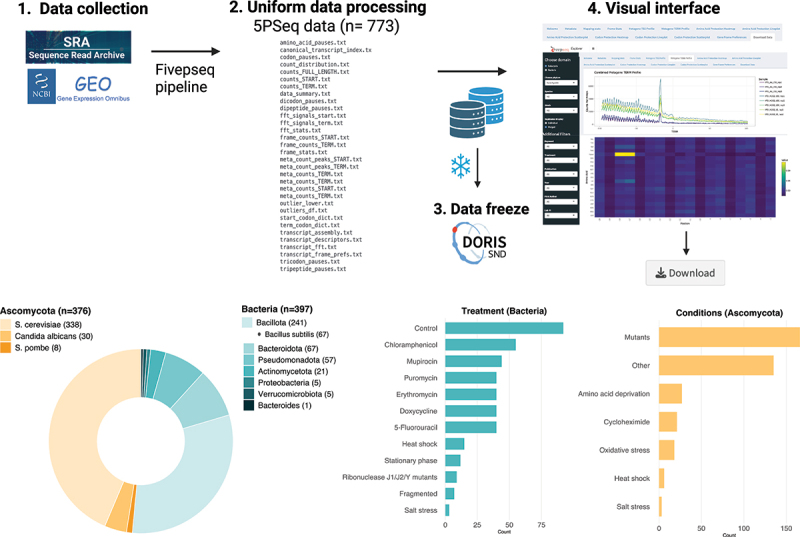
Workflow. 773 5PSeq datasets investigating cross-talk between mRNA decay and ribosome dynamics across various perturbations were curated from SRA and GEO (1) pre-processed are uniformly pre-processed (2) using the 5PSeq pipeline in python. A data freeze (3) of raw count files and statistics was created and deposited at Swedish National data Service (researchdata.Se). (4) visual explorations of the data is possible via 5PSeq-explorer (fivepseq-explorer.serve.scilifelab.se/app/fivepseq-explorer), a Shinyapp deployed via Docker as a web-based interface and a local version for plug-and-play with local processed 5PSeq data.

To enable comparability across conditions and facilitate cross-species analysis, we processed all data uniformly (see *Methods*). The generated database is deposited at the Swedish National Data Service (SND DORIS) (https://doi.org/10.48723/zchv-5x22) consists of (1) metadata, (2) raw processed counts files of amino acid, codon, frame preferences, metagene counts around start and termination sites (Figure 1) and (3) RNA compositions.

To facilitate visual exploration of the data, we created 5PSeq Explorer (fivepseq-explorer.serve.scilifelab.se/app/fivepseq-explorer), a web application built with the Shiny framework designed to facilitate comparisons across biological conditions. Details about the samples can be found under the ‘Metadata’ tab of the 5PSeq Explorer, including the associated publication, GEO and SRA id. Biological replicates are flagged in the metadata (Supplementary Table S2) allowing users to merge replicates for easier comparisons across conditions. Raw counts are normalized to library size (counts per million) for fair comparisons. Information about sequencing depth and RNA composition are available under the ‘Mapping stats’ tab. Users may download individual samples directly from the ‘Download’ tab of 5PSeq Explorer.

To enhance usability, we also provide a local version of the software that may be used to explore local 5PSeq raw counts data as a Docker container (image available at DockerHub stevensirene/5pseq-explorer-local:v1.0).

### Metadata curation and systematic exploration of biological conditions

To enable systematic exploration of biological conditions, we adopted a hierarchical top-down metadata structure inspired by prior work [28]. This approach was conceived to capture every step of data production systematically, from sample to sequencing technical details, in increasingly granular detail. Metadata descriptors (Supplementary file 2) include information about the sample (organism, phylum, species and genotype), library preparation, technical details about sequencing (instrument) and data processing (package version). Each data entry represents a biological replicate associated with a GEO/SRA ID. Importantly, biological replicates are marked in the metadata, allowing the user to merge biological replicates belonging to each experimental condition. By using the ‘Replicates display’ sidebar, a user can toggle between seeing individual and merged biological replicates for each condition.

Users can simply browse samples in the Metadata tab and interactively select/unselect sequencing samples for comparisons. To facilitate the search for biological conditions among all the samples available, we created several filters. Biological descriptors such as ‘Keyword’ (e.g. YPD amino acid deprivation, YPD control etc.) and ‘Treatment’ (e.g. Heat shock, Trehalose) can be used to filter metadata. Additional filters for searching the metadata are ‘Publication’, ‘Lab PI’, ‘First author’ and ‘Year’. This structured approach allows users to identify condition-specific ribosome behaviours, such as stress-induced stalls, without manual data parsing.

### Visualizing ribosome dynamics with 5PSeq explorer

After selecting datasets in the ‘Metadata’ tab, users can interrogate co-translationally associated ribosome dynamics. This includes global information at the genome level, aggregated metagene information at both the gene and codon levels, as well as data on individual genes. In every instance, users can interactively view plotted values by hovering their mouse over the figures and download high-resolution PNG figures of the plots by clicking ‘save’ icon located in the upper right corner supported by the plotly package [29].

**Figure 2.**
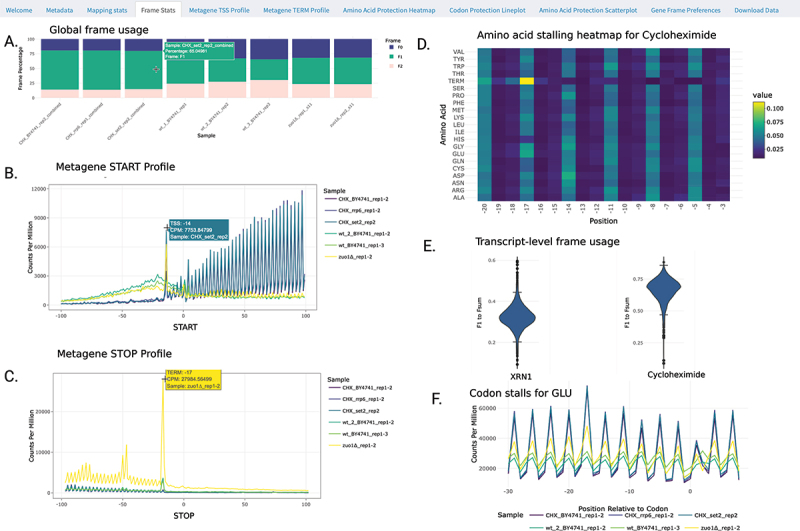
Overview of 5PSeqExplorer interface and plots. A. Frame preferences in *S. cerevisae* treated with cycloheximide (CHX, first three samples) shows a clear preference for F1 (green) compared to controls (wt samples) and *∆zuo1* mutants. B. Metagene plot around start and stop sites show ribosome stalling peaks at − 14 and −17, and a characteristic 3 nucleotide pattern corresponding to the ribosome moving one codon (3 nt) at a time. C. Lineplot of CPM counts, shows enrichment at −17 relative to codon in cycloheximide samples (blue) compared to controls (light and dark green). D. Heatmap of amino acid pauses −20 to −3 relative to all amino acids in cycloheximide treated S. cerevisiae shows strong 3 nucleotide periodicity stalling pattern (−20, −17 and −14). E. Violin plot of F1/Fsum across all transcripts (min 50 reads) shows relative frame 1 usage is preferred in cycloheximide treated BY4741 but disfavoured in *∆xrn1*cell (where there is no active 5’–3’ exonuclease trimming activity).

At genome-wide level, we provide information regarding the protection frame preferences (‘Frame stats’ tab). We sum the 5’P counts for each reading frame (F0, F1 and F2) and display their relative distributions (Figure 2A)). This enables rapid, high-level analysis of global shifts in ribosome protection. For example, Figure 2A) shows that adding cycloheximide (CHX) causes a widespread ribosome stall, slowing ribosomes, and allowing the 5’–3’ exonuclease Xrn1p to trim RNA more efficiently. As a result, there is increased 3-nt periodicity and a relative rise in 5PSeq protection at F1 (in green). We also provide a fast Fourier transform plot (FFT periodicity tab) for the observed periodicity to allow users more robust interpretations of frame dominance.

At the metagene level, we can study the relative distribution of 5P counts around the translation start and stop positions of coding genes (Metagene START and STOP tabs, Figure 2B)). 5PSeq Explorer calculates metagene Counts Per Million (CPM) coverage on the go for selected samples, enabling comparisons between different runs. However, to facilitate rigorous downstream analysis, we enable the download of (1) raw counts, (2) normalized counts for library size (counts per million) and (3) transcript-level gene frame raw counts. Metagene 5PSeq profiles are condition and species specific, but a successful *S. cerevisiae* experiment will display a typical 3-nt spike pattern (Figure 2B), blue). This reflects how ribosomes move one codon at a time, while the 5’–3’ exonuclease trims the exposed mRNA. When treated with cycloheximide, *S. cerevisiae* will typically also show a clear peak −14 nucleotides upstream of the start codon of start site (initiating ribosome) and −17 upstream of the stop codon (associated to translation termination) (Figure 2B)). Peaks at other positions might represent the ribosome stalling or even ribosome collision. For example, the double peak at −47 and −50 relative to the Stop codon represents a collision event (disome), where the trailing ribosome stalls behind another ribosome paused at termination level.

At amino acid and codon level, 5PSeq data can be explored in the tabs ‘Amino Acid Protection’ and ‘Codon protection’. Data can be visualized as line plots, heatmaps or scatterplots. Line plots allow users to focus on individual codons or amino acids across samples (Figure 2C)). Alternatively, heatmap visualization combines multiple-line plots for each sample (Figure 2D)). This offers a rapid overview of condition-specific patterns and can be particularly useful for identifying stress signatures or context-specific ribosome stalls associated to antibiotic treatment. For example, cycloheximide (Figure 2D) treatment induces global elongation arrest, causing a global increase of 3-nt periodicity. While treatment of *E. faecalis* with linezolid causes a clear context-specific ribosome stall associated to alanine that leads to an accumulation of 5PSeq reads −8 nucleotides upstream of alanine [24]. To facilitate quantitative comparison for specific features, the scatterplot tab extracts CPM coverage per amino acid and facilitates visual identification of outliers and stress-specific deviations.

Finally, under the ‘Gene Frame Preferences’ tab (Figure 2E)), users can examine the relative distribution of 5PSeq across all frames for all genes with sufficient coverage. This functionality is particularly useful for detecting deviations from the expected 3-nucleotide periodicity that reflects normal ribosome progression. For example, in *S. cerevisiae* metrics, such as *F*_1_/*F*_sum_, provide a global measure of frame preference, while ratios like *F*_1_/*F*_0_ can highlight potential frameshifting events [3]. To minimize noise and ensure robust interpretation, these plots are limited to genes with a minimum of 50 counts. We also provide a Gene Frame Ternary plot for a global overview of shifts in gene frame preference between experimental conditions (minimum 100 counts across all frames, maximum 2000 genes displayed).

### Codon-specific ribosome pauses are informative of biological stresses

**Figure 3.**
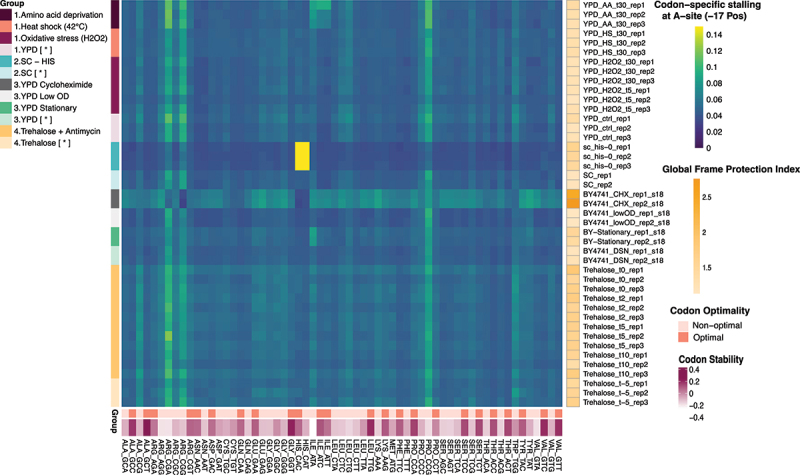
Codon specific ribosome stalls. Heatmap of codon-specific (*x*-axis) 5P counts at position −17 representing the ribosome A-site. Matched control samples from the original study are denoted by [*]. Global frame protection index (yellow heatmap) was calculated as global counts in *F*_1_/((*F*_0_ + F_2_)/2). Codon stability and optimality data are based on Presnyak et al. [30].

To highlight the value of comparing multiple 5PSeq datasets, we analysed 43 samples from eight perturbations across four independent experiments (Figure 3 and Supplementary Figure S4) and examined codon-specific ribosome pauses. As expected, histidine deprivation produced pronounced ribosome stalls at both histidine codons (CAC and CAT) (Figure 3, turquoise group), likely reflecting reduced availability of charged tRNA and impaired decoding. To provide a broader view on translation, we also considered codon optimality. Codon optimality refers to the balance between the supply of charged tRNA and their demand for translation, and it is often used as a measure of how efficiently a codon is decoded *in vivo* [30]. Optimal codons, supported by abundant tRNAs, are translated rapidly, whereas non-optimal codons with rare tRNAs slow elongation and promote ribosome stalling. Consistent with this, non-optimal codons (bottom heatmap, orange) exhibited slower translation elongation (stronger stalling) across conditions, most notably arginine (CGA and CGG) and proline (CCG). In contrast, cycloheximide treatment induced widespread stalling across all codons (grey group), consistent with its role in blocking translation elongation. This effect can also be observed when analysing the global frame protection index (Figure 3, right side) that reflects the strength of the observed 3-nt periodicity. Together, these analyses demonstrate how codon-specific stalling patterns and global frame shifts can serve as molecular signatures of nutrient stress and drug-induced translation arrest.

## Conclusion

Here we present 5PSeq Explorer (https://fivepseq-explorer.serve.scilifelab.se/), a web-based platform for interactive exploration of ribosome dynamics derived from 5P mRNA degradome sequencing (5PSeq) data. By aggregating 773 uniformly processed datasets across 23 species, we aim to address a critical gap in the systematic analysis of co-translational mRNA decay. 5PSeq Explorer is focused on changes in ribosome dynamics associated to mRNA decay that in some cases can be obscured in conventional ribosome profiling data.

5PSeq Explorer was designed to provide access to degradome data, thereby offering insights into ribosome dynamics coupled to mRNA degradation. However, since not all translation is coupled to mRNA degradation, 5PSeq Explorer is limited in its scope. The platform complements existing ribosome profiling databases, such as Riboseq.org [8], TranslatomeDB [9] and RPFdb (Ribosome Profiling Database) [10] to provide a view of global translation.

An important limitation for 5PSeq is that it reports the *in-vivo* presence of 5’ P mRNA degradation intermediates independent of its origin. In the cases where a 5’–3’ exonuclease (e.g. *Xrn1* in eukaryotes or *Rnase J* in some bacteria) trim mRNA co-translationally it can report *in-vivo* ribosome protection patterns. However, as it only studies the subsets of mRNA undergoing degradation, it should not be used to study global translation rates (as intact mRNAs undergoing translation cannot be quantified by 5PSeq). 5’ P mRNA degradation has been shown to be useful to study ribosome dynamics in yeast and plants. However, preliminary data from our laboratory (Hull *et* *al.* in preparation) suggests that the relationship between *in-vivo* 5’ P sites and ribosome protection is more complex in other eukaryotes (human, mouse or fly).

The platform offers multiple layers of analysis, from global frame usage and metagene profiles to codon- and amino acid-specific stalling patterns. These features allow researchers to interrogate translation dynamics under diverse conditions, such as nutrient stress or antibiotic treatment, and to identify molecular signatures of translational control. Beyond visualization, 5PSeq Explorer promotes reproducibility and accessibility through a public web interface (stevensirene/fivepseq-explorer:v1.7) and a Docker-based local version designed to be used with user-provided 5PSeq count files (stevensirene/5pseq-explorer-local:v1.0). This enables both interactive exploration and advanced downstream analyses. The accompanying data freeze ensures long-term availability of raw counts and metadata for computational studies. Together, these resources establish a foundation for integrative research on translation and mRNA decay.

Future developments will focus on expanding the platform with additional datasets and functionalities. Planned features include automated detection of ribosome collision signatures and integration with codon optimality metrics and ribosome profiling data, further enhancing the utility of 5PSeq Explorer for the research community.

## Methods

### Implementation

5PseqExplorer was written in R using Rshiny [31] and ShinyDashboard [32] and incorporates several R packages for dynamic data manipulation and interactive visualization (plotly (29), ggplot2 [33]). The application takes as input a folder structure containing raw counts files (in text format) obtained from the FivePseq package [27] output (Figure 1) and incorporates several normalizations steps (counts per million, merging biological replicates) and several plotting functions such as metagene plots at transcription start and end sites, heatmaps of normalized counts per amino acid and codon. The source code for the 5PSeq Explorer web-resource and local version can be found at GitHub at github.com/irenestevens8/5Pseq-Explorer. To ensure reproducibility, the Shiny application is distributed as a Docker image (stevensirene/fivepseq-explorer:v1.7) and may be run locally or accessed publicly via the SciLifeLab Serve platform. A version of 5PSeq Explorer-local is available from DockerHub (stevensirene/5pseq-explorer-local:v1.0) for use on local machine with data freeze or local 5PSeq data.

### Data collection and processing

Publicly available datasets (Supplementary Table S1) were downloaded from SRA by BioProject ID using sratools version 3.0.7 using commands prefetch and fasterq-dump. Raw data were quality assessed using FastQC v0.12.1 [34] and MultiQC v1.22.2 [35]. Next, data preprocessing was carried out in five steps: (1) adapter trimming with cutadapt 4.8 [36], (2) quality checks of trimmed data (FastQC, MultiQC), (3) UMI extraction [37], (4) mapping to reference using STAR (version: 2.7.11a options – alignEndsType Extend5pOfRead1; outFilterMatchNminOverLread 0.9; outFilterMultimapNmax 3; limitBAMsortRAM 100,000,000,000; alignIntronMax 2500) and (5) read deduplication (UMI-tools). *S. cerevisae* samples were mapped to SGD R64-1–1 (dna.toplevel.fa) and RNA content was assessed using filtered R64-1–1.103 GFF3 annotation file (where we removed transposable_element, pseudogene, SRP_RNA, RNase_P_RNA, RNase_MRP_RNA SRP_RNA, RNase_P_RNA, RNase_MRP_RNA). For *S. pombe*, genome assembly ASM294v2 (dna.toplevel.fa) and gene annotation file in GFF3 format from Ensembl were used. For studies 4, 17, 18 (Table S1), read 2 was discarded since our goal was only to look at the 5’ ends. For study 10 (Table S1), technical replicates were combined using command ‘cat SRR8756997.fastq SRR8756998.fastq SRR8756999.fastq SRR8757000.fastq > CHX_BY4741_rep1_combined.fastq’ for GSM3680765, and similarly for GSM3680766-70. For studies 14, 15 and 17 (Table S1), technical replicates were merged similarly to above. Data processing was performed using the FivePseq package version 1.3.5 using python 3.8.7. We do not apply any offset, the signal represents raw 5PSeq read counts. Bacteria 5Pseq processed data was obtained directly from the publication (https://doi.org/10.17044/scilifelab.22284709). Even though the counts files for bacteria were obtained from the Scilife Lab Data Center, these were previously processed in the laboratory using the same FivepSeq pipeline (27), making the data uniform. Codon optimality and stability data were obtained from Zhang et al. [3], based on Presnyak et al. [30].

### Data integration

Data were curated manually and harmonized through standardized metadata. All datasets in our collection originate from published studies. We verified data quality independently using FastQC and MultiQC before including it in our collection. When performing integrative analyses of the data, we recommend several steps to overcome data heterogeneity. First, we recommend using a minimum threshold for the number of reads. We provide the ‘Mapping stats’ tab for a quick view of the sequencing depth. Second, we recommend separating data by protocol version (i.e. 5PSeq should not be analysed together with newer HT-5PSeq). Since a uniform reference biological sample processed in an uniform way across laboratories is not available, we have not conducted any batch correction.

## Structured metadata

To facilitate visualization of 773 of data records available, we were selective in the metadata description (Supplementary File 2). Thus, we included the most important information about the biosample (organism, taxon, genotype and treatment) and sequencing information (platform). Entries are grouped by the title of the study (Table S1). The library preparation protocol for 5Pseq includes several variations (i.e. use of an oligo) and this information is noted in the metadata. All records are accompanied by a GEO, SRA, and PubMed ID where users should look up the full biosample record for experimental details. The metadata fields include Publication (Pubmed/MEDLINE ID), Year, First Author, Lab PI, Sample (name of biosample), Description, GEO ID, SRA ID, Title (Title of study as described in GEO), SRA genotype, Instrument, Library (library preparation protocol used as HT-5Pseq as described in (25), or 5Pseq as described in (23)), Organism (flag for distinguishing yeast versus bacteria), Phylum, Species, Strain, Keyword (flag for quick metadata description of sample), Treatment (flag for quick description of treatment condition, i.e. control vs. experimental condition), PackageVersion (Fivepseq package version used for data processing) and ReplicateGroup (flag for marking biological replicates under the same experimental condition). We also included an RNA composition file containing the following fields: Sample (name of biosample), RNA_type (Type of RNA (rRNA, mRNA etc.). A higher mRNA percentage is desirable and implies an effective rRNA depletion step) and Counts (Raw counts (not normalized to library size) of each type of RNA)

## Supplementary Material

Supplemental Material

## Data Availability

The degradome data freeze is available via the Swedish National Data Service (NSD Doris) at researchdata.se [https://doi.org/10.48723/zchv-5x22]. When reusing data, please cite (56). The source code is available via GitHub. A Docker [38] container is available for testing and deployment for the web version of 5PSeq Explorer [DockerHub stevensirene/fivepseq-explorer:version 1.7] and 5PSeq Explorer-local version (stevensirene/5pseq-explorer-local:v1.0).

## Availability of supporting source code and requirements

Project name: 5Pseq Explorer

Project home page: https://fivepseq-explorer.serve.scilifelab.se/

Operating system(s): Platform independent

Programming language: R

Licence: MIT

RRID: SCR_027718

The source code is available under MIT licence at https://github.com/irenestevens8/5Pseq-Explorer.

Documentation is available at: https://5pseq-explorer-docs.readthedocs.io/en/latest/

A Docker container is available for deployment at DockerHub (stevensirene/fivepseq-explorer:v1.7) and for 5PSeq Explorer-local version (stevensirene/5pseq-explorer-local:v1.0).

## Data freeze

The processed data from 5Pseq experiments together with sample descriptions (metadata) and RNA composition file was frozen and will be hosted on the Swedish National Data Service (SND DORIS) (https://doi.org/10.48723/zchv-5x22).
