# Supplementary material for: 5PSeq Explorer: interactive analysis of co-translational mRNA decay and ribosome dynamics
Source: RNA Biol. 2026 Mar 6;23(1):1–10. doi: 10.1080/15476286.2026.2639616 (PMC12990929; doi:10.1080/15476286.2026.2639616)
Supplement: Supplemental Material [file KRNB_A_2639616_SM9294.zip › Supplementary Table 1.docx]

**Supplementary Table 1:** Public datasets used in this study.

| **Ref** | **GEO accession** | **Title** | **BioProject ID** |
| --- | --- | --- | --- |
| [39] | GSE267940 | The early transcriptional and post-transcriptional responses to fluconazole in sensitive and resistant Candida albicans (5P-Seq) | [PRJNA1113676](https://www.ncbi.nlm.nih.gov/bioproject/PRJNA1113676) |
| [40] | GSE216524 | Analysing the effect of cellular energy levels on codon-specific ribosome occupancy in vivo | [PRJNA894167](https://www.ncbi.nlm.nih.gov/bioproject/PRJNA894167) |
| [41] | GSE193992 | Study of the cellular component-dependent functions of Xrn1 | [PRJNA798697](https://www.ncbi.nlm.nih.gov/bioproject/PRJNA798697) |
| [42] | GSE125409 | Transient transcriptome sequencing (TT-Seq) and 5'P-Seq of ATP-analog sensitive Kin28 budding yeast | [PRJNA516282](https://www.ncbi.nlm.nih.gov/bioproject/PRJNA516282) |
| [43] | GSE193912 | Not1 and Not4 inversely determine mRNA solubility that sets the dynamics of co-translational events | [PRJNA798390](https://www.ncbi.nlm.nih.gov/bioproject/PRJNA798390) |
| [44] | GSE229473 | 5P-Seq analysis of wt and new1-knockout yeast strain as well as New1:80S pull-down samples | [PRJNA954726](https://www.ncbi.nlm.nih.gov/bioproject/PRJNA954726) |
| [45] | GSE151632 | A functional connection between translation elongation and protein folding at the ribosome exit tunnel in Saccharomyces cerevisiae | [PRJNA636675](https://www.ncbi.nlm.nih.gov/bioproject/PRJNA636675) |
| [46] | GSE168290 | NOT proteins in co-translation decay | [PRJNA706755](https://www.ncbi.nlm.nih.gov/bioproject/PRJNA706755) |
| [47] | GSE128599 | Effect of cycloheximide in the co-translation mRNA degradation pattern in set2D and rrp6D strains | [PRJNA528276](https://www.ncbi.nlm.nih.gov/bioproject/PRJNA528276) |
| [45] | GSE114899 | Study of ribosome dynamics of rpl3[W255C] and its interactions with ribosome-associated chaperones | [PRJNA473001](https://www.ncbi.nlm.nih.gov/bioproject/PRJNA473001) |
| [47] | GSE118758 | Detection of cryptic start codons in set2D by 5PSeq | PRJNA486741 |
| [47] | GSE119134 | Detection of cryptic unstable transcripts associated with ribosomes in yeast | PRJNA488250 |
| [48] | GSE107250 | Study of ribosome dynamics after osmotic stress in Lsm1 and Pat1 deletions | PRJNA419414 |
| [49,27] | GSE91064 | Study of ribosome dynamics after eIF5A depletion in budding yeast | PRJNA356767 |
| [50] | GSE154212 | Molecular Functions of Conserved Developmentally-Regulated GTP-Binding Protein Drg1 in Translation | PRJNA645425 |
| [51] | GSE63120 | Widespread Co-translational RNA Decay Reveals Ribosome Dynamics | PRJNA266705 |
| [52,53] | GSE152375 | Development of High-throughput 5Pseq and detection of ribosome stalls at termination level in Saccharomyces cerevisiae | PRJNA639129 |
| [54] | GSE230202 | Ribosomes modulate transcriptome abundance via generalized frameshift and out-of-frame mRNA decay | PRJNA957826 |
| [55] | GSE153497 | Atlas of mRNA translation and decay for bacteria | PRJNA642850 |

Citations for Supplementary Table 1. Public datasets used in this study.

1. Stevens, I., Silao, F. G., Huch, S., Liu, H., Ryman, K., Carvajal-Jimenez, A., Ljungdahl, P. O., & Pelechano, V. (2024). The early transcriptional and post-transcriptional responses to fluconazole in sensitive and resistant Candida albicans. Scientific Reports, 14(1), 29012. <https://doi.org/10.1038/s41598-024-80435-w>
2. Tomaz da Silva, P., Zhang, Y., Theodorakis, E., Martens, L. D., Yépez, V. A., Pelechano, V., & Gagneur, J. (2024). Cellular energy regulates mRNA degradation in a codon-specific manner. Molecular Systems Biology, 20(5), 506–520. <https://doi.org/10.1038/s44320-024-00026-9>
3. Jordán-Pla, A., Zhang, Y., García-Martínez, J., Chattopadhyay, S., Forte, A., Choder, M., Pelechano, V., & Pérez-Ortín, J. E. (2025). Proper 5’-3’ cotranslational mRNA decay in yeast requires import of Xrn1 to the nucleus. PloS One, 20(1), e0308195. <https://doi.org/10.1371/journal.pone.0308195>
4. Tseng, S. C. (2019). Regulation of RNA Synthesis and Decay via the C-terminal Domain of Pol II. <https://asset.library.wisc.edu/1711.dl/52QPBQLOXUAEI8C/R/file-ae24a.pdf>
5. Allen, G., Weiss, B., Panasenko, O. O., Huch, S., Villanyi, Z., Albert, B., Dilg, D., Zagatti, M., Schaughency, P., Liao, S. E., Corden, J., Polte, C., Shore, D., Ignatova, Z., Pelechano, V., & Collart, M. A. (2023). Not1 and Not4 inversely determine mRNA solubility tol.bhat sets the dynamics of co-translational events. Genome Biology, 24(1), 30. <https://doi.org/10.1186/s13059-023-02871-7>
6. Turnbull, K., Paternoga, H., von der Weth, E., Egorov, A. A., Pochopien, A. A., Zhang, Y., Nersisyan, L., Margus, T., Johansson, M. J. O., Pelechano, V., Wilson, D. N., & Hauryliuk, V. (2024). The ABCF ATPase New1 resolves translation termination defects associated with specific tRNAArg and tRNALys isoacceptors in the P site. BioRxiv : The Preprint Server for Biology. <https://doi.org/10.1101/2024.05.29.596377>
7. Rodríguez-Galán, O., García-Gómez, J. J., Rosado, I. v, Wei, W., Méndez-Godoy, A., Pillet, B., Alekseenko, A., Steinmetz, L. M., Pelechano, V., Kressler, D., & de la Cruz, J. (2021). A functional connection between translation elongation and protein folding at the ribosome exit tunnel in Saccharomyces cerevisiae. Nucleic Acids Research, 49(1), 206–220. <https://doi.org/10.1093/nar/gkaa1200>
8. Allen, G. E., Panasenko, O. O., Villanyi, Z., Zagatti, M., Weiss, B., Pagliazzo, L., Huch, S., Polte, C., Zahoran, S., Hughes, C. S., Pelechano, V., Ignatova, Z., & Collart, M. A. (2021). Not4 and Not5 modulate translation elongation by Rps7A ubiquitination, Rli1 moonlighting, and condensates that exclude eIF5A. *Cell Reports*, *36*(9), 109633. <https://doi.org/10.1016/j.celrep.2021.109633>
9. Wei, W., Hennig, B. P., Wang, J., Zhang, Y., Piazza, I., Pareja Sanchez, Y., Chabbert, C. D., Adjalley, S. H., Steinmetz, L. M., & Pelechano, V. (2019). Chromatin-sensitive cryptic promoters putatively drive expression of alternative protein isoforms in yeast. Genome Research, 29(12), 1974–1984. <https://doi.org/10.1101/gr.243378.118>
10. Garre, E., Pelechano, V., Sánchez Del Pino, M., Alepuz, P., & Sunnerhagen, P. (2018). The Lsm1-7/Pat1 complex binds to stress-activated mRNAs and modulates the response to hyperosmotic shock. PLoS Genetics, 14(7), e1007563. <https://doi.org/10.1371/journal.pgen.1007563>
11. Pelechano, V., & Alepuz, P. (2017). eIF5A facilitates translation termination globally and promotes the elongation of many non polyproline-specific tripeptide sequences. Nucleic Acids Research, 45(12), 7326–7338. <https://doi.org/10.1093/nar/gkx479>
12. Zeng, F., Li, X., Pires-Alves, M., Chen, X., Hawk, C. W., & Jin, H. (2021). Conserved heterodimeric GTPase Rbg1/Tma46 promotes efficient translation in eukaryotic cells. Cell Reports, 37(4), 109877. <https://doi.org/10.1016/j.celrep.2021.109877>
13. Pelechano, V., Wei, W., & Steinmetz, L. M. (2015). Widespread Co-translational RNA Decay Reveals Ribosome Dynamics. Cell, 161(6), 1400–1412. <https://doi.org/10.1016/j.cell.2015.05.008>
14. Zhang, Y., & Pelechano, V. (2021b). High-throughput 5’P sequencing enables the study of degradation-associated ribosome stalls. Cell Reports Methods, 1(1), 100001. <https://doi.org/10.1016/j.crmeth.2021.100001>
15. Zhang, Y., & Pelechano, V. (2021a). Application of high-throughput 5’P sequencing for the study of co-translational mRNA decay. STAR Protocols, 2(2), 100447. <https://doi.org/10.1016/j.xpro.2021.100447>
16. Zhang, Y., Nersisyan, L., Fürst, E., Alexopoulos, I., Santolaria, C., Huch, S., Bassot, C., Garre, E., Sunnerhagen, P., Piazza, I., & Pelechano, V. (2025). Ribosomes modulate transcriptome abundance via generalized frameshift and out-of-frame mRNA decay. Molecular Cell, 85(10), 2017-2031.e7. <https://doi.org/10.1016/j.molcel.2025.04.022>
17. Huch, S., Nersisyan, L., Ropat, M., Barrett, D., Wu, M., Wang, J., Valeriano, V. D., Vardazaryan, N., Huerta-Cepas, J., Wei, W., Du, J., Steinmetz, L. M., Engstrand, L., & Pelechano, V. (2023). Atlas of mRNA translation and decay for bacteria. Nature Microbiology, 8(6), 1123–1136. <https://doi.org/10.1038/s41564-023-01393-z>
