# Supplementary figures and images for: 5PSeq Explorer: interactive analysis of co-translational mRNA decay and ribosome dynamics
Source: RNA Biol. 2026 Mar 6;23(1):1–10. doi: 10.1080/15476286.2026.2639616 (PMC12990929; doi:10.1080/15476286.2026.2639616)

Codon-specific stalling at A-site (-17 Pos) Control-Normalized

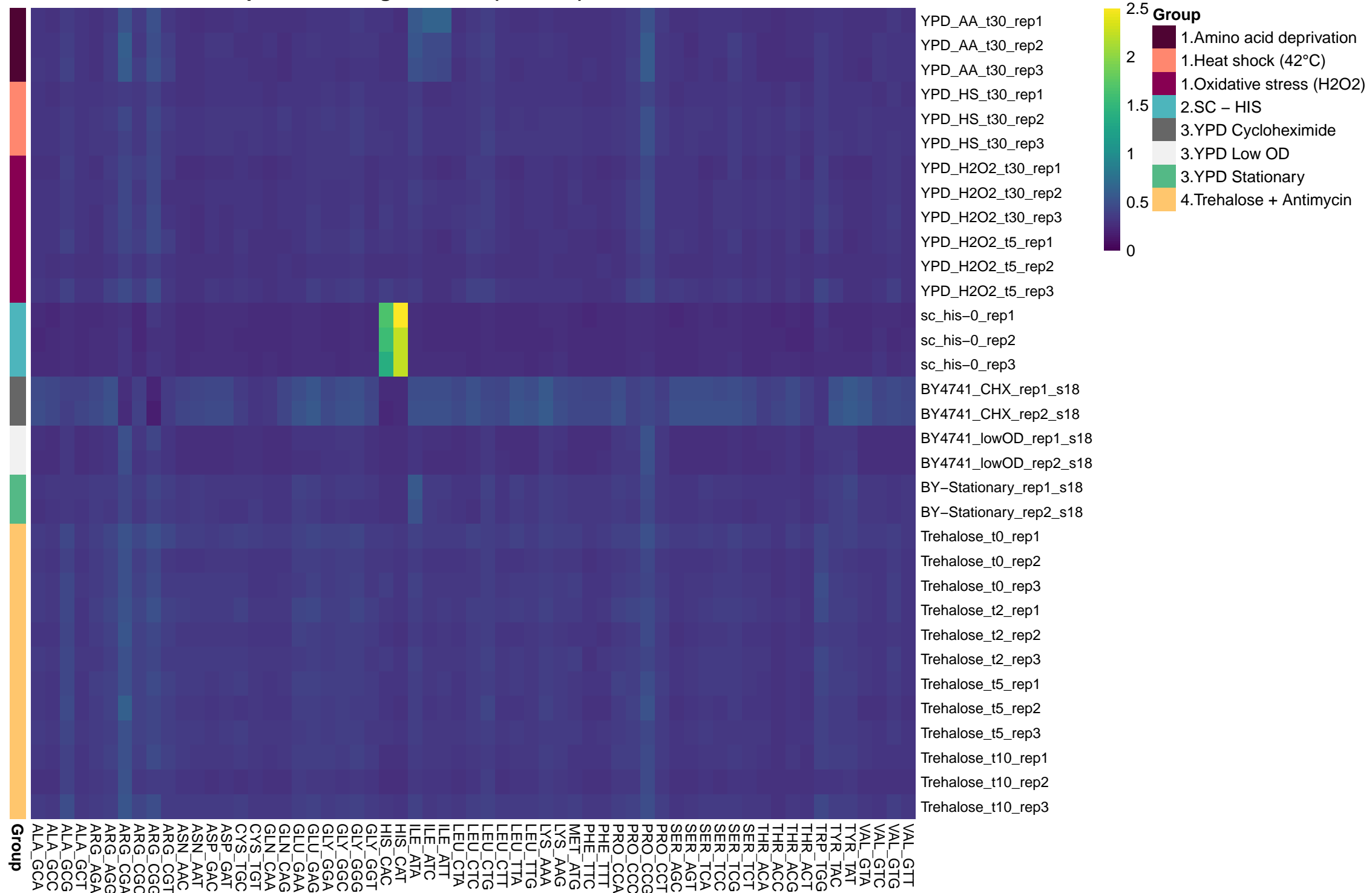

Supplement: Supplemental Material [file KRNB_A_2639616_SM9294.zip › Supplementary Figure 1.pdf]
